# Enhancing Vaccine Uptake Among Persons With Disabilities: Insights From Ghana and Implications for the Philippines

**DOI:** 10.1002/hsr2.70386

**Published:** 2025-01-27

**Authors:** John Patrick C. Toledo

**Affiliations:** ^1^ Department of Theology and Religious Education De La Salle University Manila Philippines

## Conflicts of Interest

By taking all necessary precautions, the researcher will attest that no threats were found in the research.

Dear Editor,

I've read with interest the original research entitled “COVID‐19 Vaccine Uptake and Associated Factors Among Persons With Disabilities in Ghana's Ashanti Region,” highlighted in the study conducted in Ghana's Ashanti Region by Abdul‐Aziz Seidu, Irene G. Ampomah, and Theophilus I. Emeto.^1^ This study used a cross‐sectional survey of 250 individuals in the Atwima Mponua District to evaluate the factors impacting vaccine uptake among PwDs. With a noteworthy acceptance rate of 71.2% among respondents, the researchers discovered a number of important factors influencing vaccination uptake, including age, educational attainment, disability type, and knowledge of COVID‐19.

Given that PwDs are frequently overlooked in health research, this study's scientific contribution is noteworthy because it addresses a major gap in the body of knowledge regarding the acceptance of the COVID‐19 vaccine. The authors offer empirical proof of the particular difficulties encountered by this group by employing systematic sampling and reliable data analysis techniques, such as logistic regression. The results highlight the need for specialized public health initiatives that target the particular obstacles to vaccination use, like disinformation and accessibility problems.

This research holds vital relevance to the Philippine context, where people with disabilities face barriers to basic health care.^2^ Although the Philippines has made progress in creating rules to meet the needs of people with disabilities, these policies are still not always implemented consistently. When developing initiatives to increase vaccine uptake among their own disabled community, officials in the Philippines should take into account the elements that influence vaccination acceptability that were discovered in the Ghanaian context, such as education and trust in healthcare systems.

However, this study supports inclusive vaccine awareness initiatives that successfully inform and reach people with disabilities; additionally, vaccine efficacy, safety, and accessibility are important in determining people's choices. Insights from Ghana's experience could help guide public health initiatives in the Philippines to improve vaccine accessibility and acceptance among people with disabilities, which would ultimately improve health outcomes and lessen inequities during medical emergencies.

In conclusion, ensuring inclusive healthcare for persons with disabilities is essential, as they are integral members of society deserving of equitable access to health services. By focusing on their unique needs and barriers, we can foster a more just healthcare system that honors the dignity and rights of all individuals.

## Author Contributions

**John Patrick C. Toledo:** conceptualization, methodology, and writing, original draft.

## Ethics Statement

Ethical standards are followed in the research.

## Data Availability

The data that supports the findings of this study is available from the Health Sciences Reports, and other relevant materials.
